# Indo-cyanine green administration to identify loss of lymph after axillary lymph node dissection

**DOI:** 10.3389/fonc.2023.1045495

**Published:** 2023-03-13

**Authors:** Mirela Mariana Roman, Pauline Delrue, Clarence Karler, Véronique Del Marmol, Pierre Bourgeois

**Affiliations:** ^1^ Department of Mammo-Pelvic Surgery, Institute Jules Bordet, Université Libre de Bruxelles, Brussels, Belgium; ^2^ Department of Anesthesia-Algologia Hospital Moliere, Université Libre de Bruxells, Brussels, Belgium; ^3^ Service of Dermatology, Hospital Erasme, Université Libre de Bruxelles, Brussels, Belgium; ^4^ Services of Nuclear Medicine, Institut Jules Bordet and Hôpitaux Iris Sud- Iris Ziekenhuizen Zuid (HIS-IZZ) Hospitals, Université Libre de Bruxelles, Brussels, Belgium; ^5^ Multi-disciplinary Clinic of Lymphology, Institute Jules Bordet, Université Libre de Bruxelles, Brussels, Belgium; ^6^ Service of Vascular Surgery, Institut Jules Bordet and Hôpitaux Iris Sud- Iris Ziekenhuizen Zuid (HIS-IZZ) Hospitals, Université Libre de Bruxelles, Brussels, Belgium

**Keywords:** ICG, breast cancer, axillary surgery, lymphocele, lymphatic leak

## Abstract

**Background:**

Near infrared fluorescence imaging with Indocyanine Green (ICG) is now used for the imaging of lymph nodes and lymphatic vessels. In this work, we investigated the impact of its pre-operative and peri-operative administration on our ability to detect axillary lymphatic loss after breast cancer surgery.

**Methods:**

One subcutaneous injection of ICG was administered in the ipsilateral hand of 109 women who were scheduled to have either a mastectomy with total axillary lymph node dissection (CALND) or a lumpectomy with selective lymphadenectomy (SLN) the day before (n = 53) or the same day of surgery (n = 56). The lymph leakages were assessed by means of the application of a compress in the operated armpit and by the presence or absence of fluorescence on it, as well as in the post-operative axillary drains.

**Results:**

The compress was fluorescent in 28% of SLN patients and 71% of CALND patients. The liquids in the axillary drains were also fluorescent in 71% of patients with CALND. No statistical significance was observed between the ICG injection groups. The association between compressive fluorescent and the presence of fluorescence in the axillary drains is significant in the pre-operative subgroup and in the whole group.

**Conclusion:**

Our research demonstrates that lymphatic leaks aid in the development of seromas and calls into question the effectiveness of the ligatures and/or cauterizations used during surgery. A prospective, multicentric, randomized trial should be conducted to verify the efficacy of this approach.

## Introduction

Axillary lymph node dissection represents the standard surgical treatment for breast cancer patients with clinically node-positive axilla. SLN biopsy has rapidly replaced standard axillary lymph dissection as the choice of several surgeons, and it is considered the standard procedure for axillary evaluation in early-stage breast cancer (1). However, it is associated with several problems like axillary web syndrome, arm lymphedema, neuropathy, hematoma, infection, and seroma (2–4).

Currently, one recommended approach for preventing problems like arm lymphedema is the “axillary reverse mapping” (ARM) procedure (5). Of several tracers, Indocyanine Green (ICG) has been proposed for such an approach (6, 7) and for sentinel lymph node imaging (8).

In previous work (9), we first studied if there was one difference in our ability to detect the fluorescence of the lymph nodes draining the arm in cases of complete axillary lymph node dissection (CALND) and/or SLN biopsy when ICG was injected in the first interdigital space of the hand either the day before (pre-operative) or the same day (peri-operative), and we observed no statistical difference.

The aim of the current study is to describe our ability to detect fluorescent lymph leakages during surgery at the level of the axillary walls because of such pre- and per-operative injections of ICG, as well as to determine post-surgery the continuous existence of such fluorescent lymphatic liquids in the collected liquids (in the drains).

## Materials and methods

This prospective monocentric study was approved by the Investigational Review Board (IRB) of the Jules Bordet Institute (CE2876) and registered in the European Clinical Trials Database (EudraCT number 2018-002862-38).

After giving written, signed informed consent, 109 women who were scheduled to have a mastectomy with complete axillary node dissection, a lumpectomy with selective lymphadenectomy (SLN), or a lumpectomy with complete axillary node dissection (CALND) for a mammary tumor that had been confirmed histologically were subsequently enrolled in the study between June 2019 and June 2021.

Our statistician determined these base values “*a priori*.”

Exclusion criteria were: (1) history of iodine allergy or anaphylactic reactions to insect bites or medication; (2) hyperthyroidism; (3) severe cardiac or pulmonary disease; (4) significant renal failure and (5) pregnancy. Patients were not limited in their normal behavior, diet, or medication intake before the study.

ICG (0.2 ml from 25 mg of ICG diluted by 5.0 ml of sterile water for injection) was injected subcutaneously in the first interdigital space of the hand of the operated side, either the day before the operation or just before the operation (at the induction of anesthesia or within half an hour before), depending on the patient’s hospitalization time.

For the SLNB identification, radio-colloids were injected in the breast and around the tumor the day before surgery and before the injection of ICG. In the operating room, were removed as sentinel all the LN seen on pre-operative lymphoscintigram and with a signal higher than 10% of the most active one (10).

For patients who underwent a standard CALND, at least levels I–II were removed.

A specialized near-infrared camera system (PDE, Hamamatsu) is used for fluorescence imaging. A light-emitting diode light source set to a wavelength of 760 nm is used, and the detector is a charge-coupled device (CCD) camera with a filter set to detect light with a wavelength of 820 nm. The fluorescent signal is sent to a digital video processor to be displayed on a monitor in real time. The camera was held directly by the surgeon at a standard distance from the operative specimen.

Our near-infrared camera system identified fluorescent axillary nodes in the operating room; these nodes were classified as ARM nodes.

**Figure 1:**
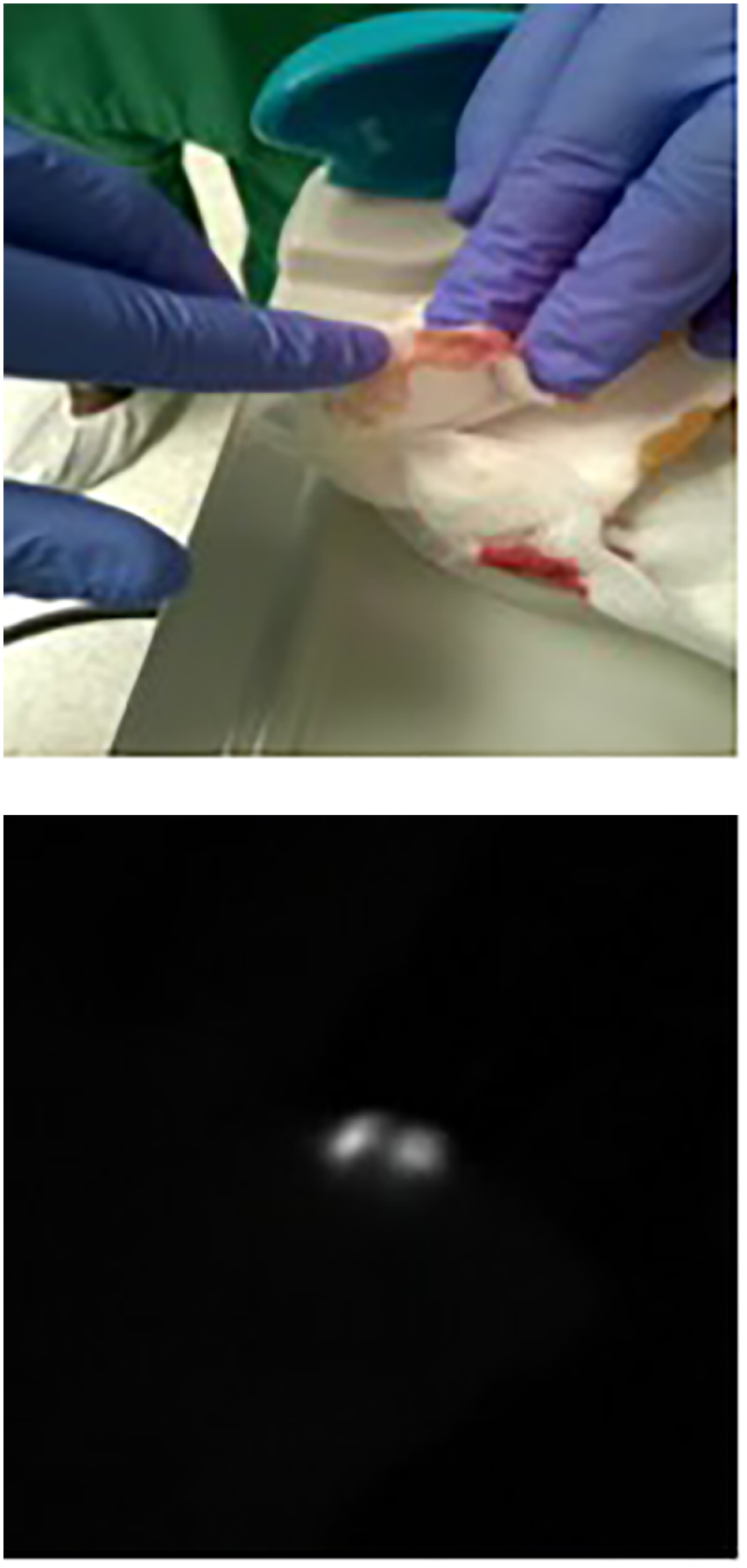
The “touch-and-view approach” (fluorescence of compress). The index finger of the hand shows the presence of fluorescence on the compress put in the axilla after the end of axilla surgery and as controlled by the NIRFI system as seen in the right-side black-and white picture.

After axillary lymph node dissection and before closing, a compress was put inside the axilla to verify lymphatic loss. If fluorescence was detected on the compress using our near-infrared camera system (see **Figure 1**), it was considered positive for lymphatic loss. At the end of surgery, a drain was placed in the axilla after CALND, and a lumpectomy was done in the upper and outer quadrants close to the incision for SNL biopsy.

**Figure 2:**
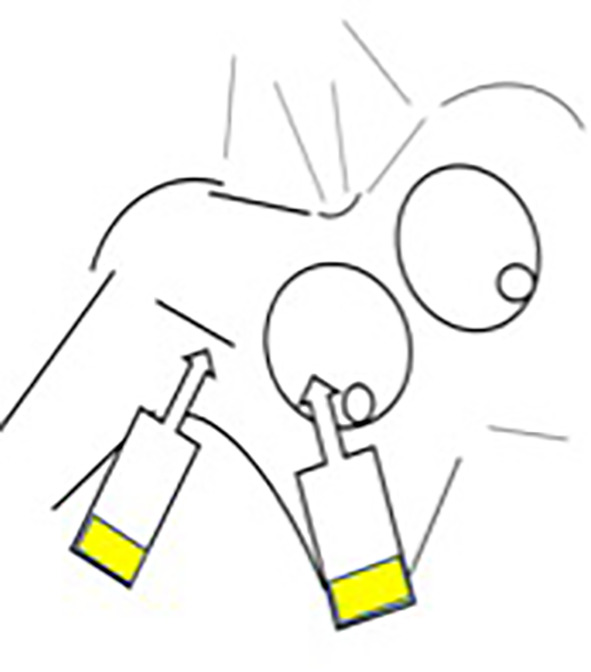
Fluorescence in the drains after surgery (during hospitalization). Fluorescence of the axillary drain; see **Figure 3**. Fluorescence of the mammary drain: see **Figure 4**

**Figure 3:**
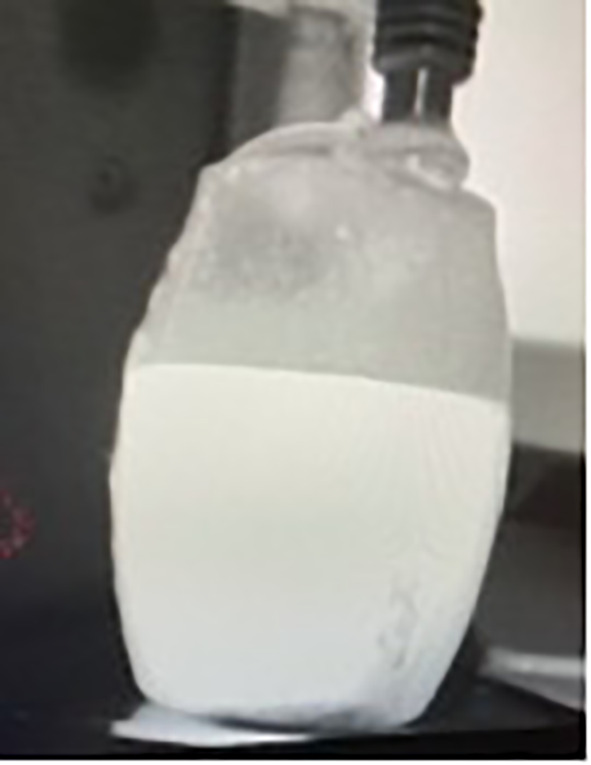
Fluorescence of axillary drain.

**Figure 4:**
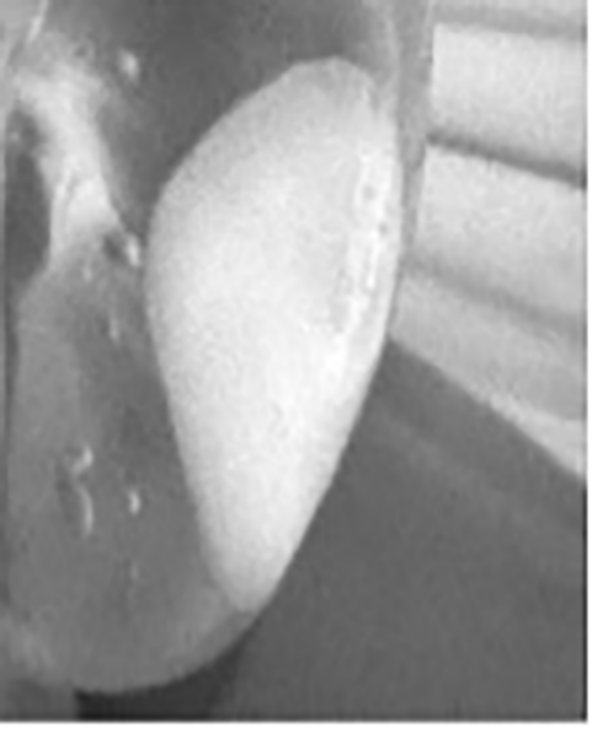
Fluorescence of mammary drain.

After surgery, we recorded and analyzed drainage volumes and liquid fluorescence (or lack thereof) using our near-infrared camera system. The drain was then removed when fluid production was less than 50 ml per day (**Figures 2**–**4**).

Each patient was seen one week after surgery and then weekly or more frequently.

The outcomes were defined as the potential differences between pre- and peri-operative injections of ICG in the ipsilateral limb for patients who undergo a CALND:

– on our ability to detect per-operatively the loss of fluorescence lymph by one touch-and-view approach using a compress put in the axilla,
– on the fluorescence or not of the liquids collected in the drains.

### Statistical analysis

Our analysis is based on a set of statistical tests to assess the impact of temporal variation in ICG administration on the main parameters. The parameters of interest were divided into categorical variables (fluorescence or not of the SLN biopsy, fluorescence or not of the axillary LN in the CALND, pN+ status) and quantitative variables (number/percentage of fluorescent lymph nodes in the CALND). The standard Chi-Square test was used to assess whether the categorical parameters depend on the timing of ICG injection. However, when the number of patients in each category is too small, the Fisher exact test is used. On the other hand, the Kruskal–Wallis non-parametric test was used to determine if the distributions of the quantitative variables were different depending on the moment of the ICG injection. A nonparametric method was applied when the Shapiro–Wilk test led to the rejection of the normality hypothesis. All statistical analyses were carried out using the R software. We consider 0.05 as the level of significance for all statistical tests (11).

## Results

Between June 2019 and June 2021, 109 women (mean age = 61, range 23–93 years) were successively enrolled in the study who were scheduled to undergo either lumpectomy (n = 54) with selective lymphadenectomy (SLN) (mean age = 62.1, range 28–93 years), mastectomy (n = 20) with complete axillary node dissection, or lumpectomy (n = 35) (mean age 59.9, range 23–89 years) with complete axillary node dissection (CALND) for a histologically proven mammary tumor.

The characteristics of patients are shown in **Tables 1**, **2**. The SLN biopsy patients in group A (injected pre-operatively) did not differ statistically from the patients in group C (injected peri-operatively) as well as the CALND patients in group B (injected pre-operatively) from the ones in group D (injected peri-operatively).

**Table 1:** Characteristics of patients with pre-operative and per-operative injection of ICG.

	Pre-operative injection of ICG		Peri-operative injection of ICG			
	SLNB (A)	CALND (B)	SLNB (C)	CALND (D)	A *vs* C	B *vs* D
**No. of patients**	28	26	26	29	*p*	*p*
Age						
** Median**	60.7	58.9	63.5	60.9	*ns*	*ns*
** Range**	28–83	39–89	35–93	23-85		
BMI						
** Median**	27.1	28.5	26.2	27.5	*ns*	*ns*
** Range**	19.8–40	17.4–41	18.5–40.2	18.6–41		
Lateralization						
** Left**	13	17	16	17	*ns*	*ns*
** Right**	15	9	10	12		
Tumor size, mm						
** Median**	17.14	34.2	18	33.72	*ns*	*ns*
** Range**	4–70	10–70	8–30	8–87		
**Histology**						
** IDC**	25	21	25	27	ns	ns
** ILC**	3	5	1	2		
Molecular classification						
** Luminal A**	7	2	1	0		
** Luminal B**	15	15	18	13		
** HER2-enriched**	1	1	1	9		
** Triple negative**	5	6	6	7	*ns*	*ns*
Grade						
** 3**	7	10	12	16		
** 2**	13	12	6	12	*ns*	*ns*
** 1**	8	4	8	1		

ICG, indocyanine green; SLNB, sentinel lymph node biopsy; CALND, complete axillary lymph dissection; BMI, body mass index; IDC, invasive ductal carcinoma; ILC, invasive lobular carcinoma.

ns, not statistically signifcant.

**Table 2:** Characteristic of surgery and drains in patients with pre-operative injections of ICG (pre-op inj. of ICG) and in patients with peri-operative injection of ICG (peri-op inj. of ICG).

Type of surgery		SLNB (A)	CALND (B)	SLNB (C)	CALND (D)	
	**Lumpectomy**	28	15	26	20	
	**Mastectomy**	0	11	0	9	*ns*
**No. of lymph node resected**						
**SLNB**	**Median**	1.89		2.08		
	**Range**	1–6		1–5		*ns*
**No. of lymph node resected**						
**CALND**	**Median**		9.8		11.1	*ns*
	**Range**		4–29		5–27	
**AP Axillary status**	**pN0**	27	11	24	8	
	**pN+**	1	15	2	21	*ns*
**Drains**	**Breast + axilla**	0	5	0	5	
	**Breast only**	28	21	26	24	*ns*
	**Axilla only**	0	21	0	24	

ICG, indocyanine green; SLNB, sentinel lymph node biopsy; CALND, complete axillary lymph dissection.

Pre-op inj. of ICG Peri-op inj. of ICG. ns, not statistically signifcant.

### Peri-operative detection of axillary lymph loss through the touch-and-print (“compress”) approach

In the SLN biopsy groups, the compress was fluorescent in 16/56 (28%) cases with no statistical difference between the peri- and pre-operative groups of ICG injection, in 10/29 (34%) cases in the pre-operative injected group and in 6/27 (22%) in the peri-operative group.

The compress was more frequently found fluorescent in 38 out of 55 patients with CALND (69%) with no statistical significance between the peri- and pre-operative groups of ICG injection, in 17 out of 26 in the group pre-operative (65%), and in 21 out of 29 in the group peri-operative (72%).

### Post-operative detection of lymphatic fluorescence in the mammary drains of the SLN biopsy groups

In the SLN biopsy groups, the liquids in the mammary drains were fluorescent in 7/54 cases (13%) with no statistical difference between the peri- and pre-operative groups of ICG injection, in 5/28 cases (18%) in the pre-operative group, and in 2/26 (8%) in the peri-operative group.

### Post-operative detection of lymphatic fluorescence in the mammary drains in the CALND groups

In the CALND groups, the liquids in the mammary drains were fluorescent in 11/45 cases (24%) with no statistical difference between the peri- and pre-operative groups of ICG injection, in 4/21 cases (19%) in the pre-operative group and in 7/24 (29%) in the peri-operative group.

### Post-operative detection of lymphatic fluorescence in the axillary drains

The liquids in the axillary drains were fluorescent in 38 out of the 55 patients with CALND (71%), with no statistical significance between the peri- and pre-operative groups of ICG injection, in 17 out of the 26 in the group pre-operative (65%), and in 21 out of 29 in the group peri-operative (75%).

The liquids in the “only” axillary drains were fluorescent in 30 out of the 45 patients with CALND (67%) with no statistical significance between the peri- and pre-op groups of ICG injection, in 13 out of the 21 in the group pre-operative (62%), and in 17 out of 24 in the group peri-operative (71%).

When one fluorescence was detected post-operatively, it was observed in all the drains but decreased from day +1 to day +3.

### Peri-operative detection of axillary lymph loss through the touch-and-view (“compress”) approach versus post-operative detection of lymphatic fluorescence in the drains

In **Table 3**, we analyzed the correlation between one compress fluorescent and the presence of fluorescence in the axillary drains of patients with CALND.

**Table 3:** Relationship between per-operative detection of compress fluorescent and post-operative fluorescence in the drains.

Fluorescent Compress	Fluorescent drains after pre-operative ICG			Fluorescent drains after peri-operative ICG			Total drains fluorescent		
	Yes	No	Total	Yes	No	Total	Yes	No	Total
**Yes**	17	0	17	18	3	21	35	3	38
**No**	4	5	9	5	3	8	9	8	17
**Total**	21	5	26	23	6	29	44	11	55
***P-value***	*0.002*			*0.305*			*0.0019*		

Using the Fisher exact test, the association is significant in the pre-operative sub-group (p = 0.002) and in the whole group (p = 0.0019), but not in the peri-operative group (p = 0.305). While using the Cochran–Mantel–Haenszel test, which analyzes the correlation between the variables, the correlation appears highly significant (p = 0.003).

The positive predictive value, the negative predictive value, and the overall accuracy of one compress found fluorescent per-operative for the post-operative identification of fluorescence in the drain were:

– In the whole group, 35/38 or 92%, 8/17 or 49% and 43/55 or 78%
– in the group pre-operative, 100%, 5/9 or 55% and 22/26 or 84%
– in the group peri-operative, 85%, 18/21, 3/8 or 38% and 21/29 or 72%.

### Peri-operative detection of fluorescent axillary lymph nodes versus post-operative detection of lymphatic fluorescence in the drains

In **Table 4** below, the correlation between LN fluorescent and the presence of fluorescence in the axillary drains of CALND patients was analyzed.

**Table 4:** Relationship between peri-operative detection of fluorescent axillary lymph nodes (LN) and post-operative detection of lymphatic fluorescence in the axillary drains.

LN fluorescent	Fluorescent drains after pre-operative ICG					Fluorescent drains after peri-operative ICG			Total drains fluorescent		
	Yes	No		Total		Yes	No	Total	Yes	No	Total
**Yes**	18		1		19	20	2	22	38	3	41
**No**	3		4		7	3	4	7	6	8	14
**Total**	21		5		26	23	6	29	44	11	55
***P-value***	*0.01*					*0.018*			*0.0003*		

The positive predictive value, the negative predictive value, and the overall accuracy of one LN found fluorescent peri-operative for the post-operative identification of fluorescence in the drain were:

– in the whole group, 38/41 or 92%, 8/14 or 57%, and 44/55 or 80%
– in the group pre-operative,18/19 or 94%, 4/7 or 57%, and 22/26 or 84%
– in the group per-operative, 20/22 or 90%, 4/7 or 57%, and 24/29 or 82%.

The numbers and percentages of fluorescent LN in the axillary pieces of dissection were also not different in the pre-operative and peri-operative groups of ICG injection when the presence of fluorescence in the drains was detected or not.

## Discussion

The visualization of lymph nodes and lymphatic vessels using indocyanine green (ICG) and laser-assisted near-infrared (NIR) imaging systems is now considered an established method for the detection of sentinel lymph nodes and evaluation of lymphedema (12–14).

In the literature, the incidence of seromas varies from 10% to 85% (15), which could be due to a lack of consensus on their definitions (16). Some authors classified seromas as any palpable fluid accumulation following drain removal, while others described them as clear fluid aspirated from a palpable collection (17–23). Uncertainty also persists regarding the fundamental mechanisms of seroma development and formation (24).

Different hypotheses were expressed to explain these liquid collections, such as the acute inflammatory reaction following surgical trauma, the response to increased fibrinolytic activity in serum and lymph, the reduction of fibrinogen levels in plasma, the patient’s high body mass index, hypertension, preoperative radiation, tumor size, extended breast and axilla surgery (25–37), the use of electrocautery and the cellular damage by thermal effect, the incomplete vessels and lymph ducts obliteration during the dissection, and extended axillary lymph node involvement (38, 39), as well as lymphatic leakages collecting in the dead space following axillary dissection. The hypothesis that the fluid is of lymphatic origin was raised in the 1990s (38–40). By examining cell composition and cytokine analyses, Montalto et al. (41) said that seromas are caused by an accumulation of afferent lymph. In contrast, other studies have reported that the cell content of seroma liquid differs from lymph fluid, with a higher protein content and no fibrinogen present, more like an exudate resulting from an inflammatory reaction during the first phase of wound repair (41, 42).

In a subject that is still a matter of debate (43), our results and the presence of fluorescence in the drains after surgery show that lymphatic fluid and leakages contribute at least partially to seroma development and formation in 62% to 75% of patients.

Other tracers can be and are proposed to image the lymphatic vessels, the lymph nodes, the lymphatic leakages, and the lymphatic collections. Vital dyes, such as isosulfan blue and methylene blue, are commonly used for intraoperative identification of lymphatic leaks, but a risk of allergic reactions has been reported in 0.07 to 2.7% of cases (24, 44–46). One benefit of ICG over other lymphatic tracers is the potential for long-term detection in drains. Alternatives like radiolabeled tracers are conceivable and have the benefit of allowing quantification of the labeled molecules but are constrained by their physical half-life (6 h for 99m-Technetium and 2.8 days for 111-Indium).

In the framework of this study, we also show interest in ICG injection for the intraoperative identification of lymphatic leaks. Our “touch-and-view” approach represents the simplest way to diagnose such leakages that also can visualized in the axilla itself using near-infrared imaging systems.

Giacalone et al. have reported the use of ICG to show per-operatively the lymphatic vessels at the origin of chronic and persistent lymphocele and to perform lymphatic to vein anastomosis (LVA) and/or lymphatic ligation (47). ICG was also used by Boccardo et al. to perform LVA in patients at risk of developing lymphedema (48).

Such a confirmed ability to detect lymph leakages and their contributions to the formation of seromas raises the question of implementing per-op ligatures and/or cauterizations of the leaking vessels to prevent seroma formation with their punctures and complications.

The present study contains certain limitations. Firstly, the patient number was relatively small. Secondly, the information lacked data on patients with no injection of ICG. Therefore, a large randomized, controlled, multicenter trial should be conducted in the future to address these limitations.

To conclude, our study provides important clinical applications for patients who have had axillary surgery for breast cancer and represents the basis for such an approach. However, a prospective randomized and multicentric investigation should be conducted to confirm the interest and safety of such an attitude.

## Data availability statement

The original contributions presented in the study are included in the article/supplementary material. Further inquiries can be directed to the corresponding author.

## Ethics statement

This prospective monocentric study was approved by the Investigational Review Board (IRB) of the Jules Bordet Institute (CE2876) and was registered at the European Clinical Trials Database (EudraCT number 2018-002862-38). The patients/participants provided their written informed consent to participate in this study.

## Author contributions

BP and RM contributed to conception and design of the study. DP and RM organized the database. PB performed the statistical analysis. PB and RM wrote the first draft of the manuscript. RM, PB, and DV wrote sections of the manuscript. All authors contributed to the article and approved the submitted version.
